# Single-Nucleus RNA-Sequencing Reveals Sex-Dimorphic Molecular Remodeling by Running and TFEB-Overexpression in Skeletal Muscle

**DOI:** 10.1093/geroni/igaf122.1534

**Published:** 2025-12-31

**Authors:** Constanza Cortes

**Affiliations:** University of Southern California, Los Angeles, California, United States

## Abstract

Endurance exercise induces complex structural and functional remodeling in skeletal muscle, promoting metabolic efficiency and geroprotective effects. However, to date, our understanding of how these pathways may differ by sex remains poorly understood. We have previously shown that TFEB-overexpression in skeletal muscle (cTFEB;HSACre transgenic mice) recapitulates multiple aspects of running-activated metabolic remodeling across the lifespan. To determine the degree to which running and TFEB-overexpression transcriptionally remodels skeletal muscle, we performed single-nuclei RNA sequencing on tibialis anterior muscle from male and female mice across young sedentary, voluntary wheel running, and muscle-specific TFEB-overexpressing groups. Our results reveal significant sex- and condition-specific changes in myonuclei subtypes and non-myonuclear populations, including fiber-type switching, extracellular matrix remodeling, mitochondrial function, and immune signaling pathways. Importantly, TFEB overexpression recapitulates many transcriptional features of endurance exercise, with convergent yet distinct transcriptional responses across sexes, highlighting shared molecular pathways involved in muscle adaptation. This study advances our understanding of the molecular mechanisms driving sex-specific skeletal muscle plasticity in response to endurance running and demonstrates that TFEB overexpression effectively mimics key aspects of exercise-induced transcriptional remodeling in a sex-dependent manner. Moreover, it positions cTFEB;HSACre transgenic mice as a valuable experimental system for the discovery and prioritization of novel exercise-mimetic therapeutics aimed at promoting geroprotective muscle adaptations.

